# International patent families: from application strategies to statistical indicators

**DOI:** 10.1007/s11192-017-2311-4

**Published:** 2017-02-28

**Authors:** Antoine Dechezleprêtre, Yann Ménière, Myra Mohnen

**Affiliations:** 1 Grantham Research Institute on Climate Change and the Environment and Centre for Economic Performance, London School of Economics, London, UK; 2 MINES ParisTech, PSL – Research University, CERNA, i3 UMR CNRS, 60 boulevard Saint Michel, Paris, 75006 France; 3 University of Essex, Colchester, UK

**Keywords:** Patent families, Patent value, Priority patents, Continuations, Divisional applications, O31, O34

## Abstract

This paper provides an in-depth analysis of the characteristics of international patent families, including their domestic component. We exploit a relatively under-studied feature of patent families, namely the number of patents covering the same invention within a given jurisdiction. Using this information, we highlight common patterns in the structure of international patent families, which reflect both the patenting strategies of innovators and the peculiarities of the different patent systems. While the literature has extensively used family size, i.e. the number of countries in which a given invention is protected, as a measure of patent value, our results suggest that the number of patent filings in the priority country within a patent family as well as the timespan between the first and last filings within a family are other insightful indicators of the value of patented innovations.

## Introduction

Patents have been increasingly used as an indicator of innovation activity over the last decades, and the recent availability of global patent databases such as the European Patent Office’s Worldwide Patent Statistical Database PATSTAT has only reinforced this trend. However, it has long been acknowledged that using patent statistics to measure the productivity of research and development activities is not without limitations (Pavitt 1982, 1985; Griliches 1990). Some innovations are never patented, while others are protected by several patents. Mere patent counts tell little about the market value of patented innovations, which varies considerably. Furthermore, patent design and patenting practices differ between countries. Moreover, the propensity to patent (e.g. number of patents filed per USD of R&D) has been increasing faster in some industries and jurisdictions, making it all the more difficult to use patent metrics for comparisons across time, sectors, and/or countries (Hall and Ziedonis 2001; Jaffe and Lerner 2004; Van Zeebroeck et al. 2009).

Patent families go a good way in alleviating these methodological shortcomings. Patent families generally refer to the whole set of patents covering the same invention in one or more countries. Formally, it encompasses “the set of patents (or applications) filed in several countries which are related to each other by one or several common priority filings” (OECD 2009).^1^ Counting the number of patent families avoids double counting patents filed in several countries, thus providing a count of patent inventions (de Rassenfosse et al. 2013). Since the decision of a firm to patent in a particular country signals an intention to enter into a local market and sell a new product or use a new technology. Patent families have therefore been used to measure the international diffusion of technologies (e.g. Eaton and Kortum 1999; Dechezleprêtre et al. 2011). The size of patent families—defined as the number of countries where the family is represented—may be used to measure the invention’s market size and, hence, its value (Harhoff et al. 2003).

In this paper, we focus on another dimension of patent families: the number of patents that are part of the same family within each jurisdiction. Indeed, inventions may be patented in several countries and may be protected by several patents in each country. Moreover the number of patents protecting the invention may differ between countries.

Our objective is to carry out a comprehensive analysis of the structure of patent families that takes these dimensions into account. In contrast to recent studies which have looked at domestic patent families in a United States (Hegde et al. 2009) or European context (Harhoff 2006; Gambardella et al. 2008; van Zeebroeck and van Pottelsberghe 2011a), we take a global approach and jointly consider the domestic and international aspect of patent families in all three major patent systems: the European Patent Office, the United States Patent and Trademark Office and the Japan Patent Office. This makes it possible to shed light on statistical patterns in the structure of patent families, reflecting both the patenting strategies of innovators and the peculiarities of the different patent systems. Based on these patterns, we revisit the interpretation of statistics on patent families, and propose new international patent-based indicators enabling more rigorous cross-country comparisons of innovation performances at the national and sector levels.

Our approach is based on a statistical analysis of the global PATSTAT database between 1993 and 2010, supplemented by interviews with IP attorneys and companies and by a review of patent law in the European, American and Japanese patent systems. Our interviews suggest that patent families should be viewed from a dynamic perspective, taking into account the maturation process of innovations. Applicants indeed face a trade-off between the pre-emption of patent protection at an early stage of the innovation development, and the fine-tuning of patent protection as the innovation matures and its market potential becomes clearer. We show that national patent systems and international procedures jointly offer various types of flexibilities that allow inventors to reconcile these objectives through sequential patent applications. In fact, inventors typically file multiple applications in the first filing country (the priority country^2^) as a first step and seek protection in other countries later in the maturation process by filing a single foreign patent.

This finding has implications for patent-based statistics. In particular, we show that the number of patents filed in the priority country along with the timespan between the first and last filings within a patent family are strongly correlated with other commonly used measures of patent value such as forward citations, family size, grant status and membership of triadic and PCT families. Compared to other indicators such as the size of international families, the number of patents filed in the priority country as well as the family timespan provide common metrics for all inventions, including those that are never patented abroad, which represent 80% of patented inventions worldwide. By sorting patents according to their national family size, we argue that these measures are able to deal with the very high proportion of purely domestic patents filed for example in Japan or China. We thus refine existing indicators such as the count of priority filings proposed by de Rassenfosse et al. (2013). The timespan between the first and last filings within a patent family has the advantage of combining information from both the priority country and foreign offices and is relatively robust to administrative idiosyncrasies of the various patent offices.

The article is organized as follows. We review the literature on the use of patent statistics as indicators of innovation in the following section. We then analyse the legal and economic determinants of the structure of national and international patent families, with a specific focus on the US, European and Japanese patent systems. In fourth section we describe the methodology used to analyse patent families and provide some descriptive statistics on the composition of patent families since 1993. Fifth section presents our econometric analysis and introduces new measures of patent value based on the number of patents filed in the priority office and on the timespan of patent families between the first and the last patent applications. Sixth section compares countries’ innovative output based on different patent counts. We conclude by reflecting on the implications of the new measures presented.

## Patents as innovation indicators

Patents are one of the main indicators used to assess the productivity of innovation systems. In contrast to R&D spending, patents do not measure inputs but outputs of the R&D process. Compared with academic publications—an alternative indicator of innovative output—they are more oriented towards the industry and cover a broader scope of technologies (Freeman 1982; Grupp 1997, 1998; Frietsch and Schmoch 2006). Especially in high-technology areas, patents can help to assess present and future competitiveness of companies, sectors, or economies, since they indicate a potential for opening new markets or gaining market shares with new products (Frietsch and Schmoch 2006; Schmoch 2004). In addition, patent data are now widely available, making it possible to generate large datasets for statistical analyses without having to conduct costly interviews or surveys. They also include a wealth of information, in particular fine technology classifications allowing to conduct analysis at low levels of aggregation.

Patent-based indicators are however imperfect proxies for technological innovation. First, they are not the only way to protect inventions, as inventors may instead rely on trade secret or lead time (Cohen et al. 2000) even if the most economically significant inventions seem to have historically been patented (Dernis et al. 2001). Second, the propensity to patent differs across sectors, technological fields, and countries, depending on how patent law is enforced. Third and most importantly, it is widely recognized that the distribution of patent value is highly skewed (Scherer 1965; Pakes and Schankerman 1984; Pakes 1986; Griliches 1990). As a general rule, there is a large number of patents of limited value and a small number of highly valuable ones. Among high-value patents, the distribution is still uneven: examining German patents that have been renewed during at least 17 years, Harhoff et al. (1999) find a highly skewed value distribution, referred to as a “tail within the tail”. This means that simply counting the number of patents assuming that they were all of equal value is bound to generate significant biases in the measure of innovation.

A natural solution to this problem is to weigh patents by some indicator of their value. Various measures have been used as proxies for the unobserved monetary value of patents (for recent overviews of patent quality measures, see van Zeebroeck 2011; van Zeebroeck and van Pottelsberghe 2011b; Squicciarini et al. 2013).^3^ Generally, patent values are inferred from characteristics of either the patent itself or the patent owner. These variables are typically only loosely correlated with each other and are thus sometimes aggregated into composite patent quality indexes (Lanjouw and Schankerman 2004; van Pottelsberghe and van Zeebroeck 2008; van Zeebroeck 2011). In the remainder of this section we review the most frequently used indicators of patent value in greater detail and use them to assess the quality of our proposed new indicators in sector 5.

A first way to measure the value of patents is to resort to surveys of patent holders who are asked to provide an assessment of the private value of their patents (e.g. Harhoff et al. 2003; Gambardella et al. 2008). The major drawback of this approach is obviously the size of the sample which is naturally limited by the cost of undertake such surveys, making it unusable on a large scale. As a consequence, survey-based measures have been mostly used to show that patent value is correlated with a number of features of the patents that are readily available in patent databases.

Some value indicators are observable as soon as the patent is published. Those include the number of claims, which provides an indication of the legal breadth of patent protection and signals the complexity of a patent. Tong and Frame (1994) show that patents weighted by their claims are positively linked to other measures of national research performance, while Lanjouw and Schankerman (2001) show that a patent is more likely to be litigated if it has more claims. The number of IPC classes mentioned on the patent application is another measure of the scope or breadth of a patent. As inventions are considered to be a combination of existing ideas, the wider set of ideas, the more valuable the patent (Guellec and van Pottelsberghe de la Potterie 2000). Finally, backward citations—i.e. references to previous patents made in the patent application—have also been used as an indicator of value based on the idea that backward citations signal a patent of a larger scope. A higher number of backward citations however also causes the content of the patent to be more restricted and therefore limits its possible value (Harhoff et al. 2003), so that the relationship between backward citations and patent value can be ambiguous.

Although the above indicators have the advantage of being immediately available to the researcher, a lot of information on patent value is only observed a long time after the initial publication. The grant status of a patent is one such indicator of value since the granting process confirms the novelty, applicability and inventiveness of the invention, and confers monopoly rights to the holder (Guellec and van Pottelsberghe de la Potterie 2000). The grant status has been shown to be correlated with other measures of value (Hall et al. 2005; Lanjouw et al. 1998). A potential disadvantage of grant status is the substantial length of the process, which is aggravated by the increased workload at the patent offices and by the possibility to delay the start of the examination process in certain patent offices (Harhoff 2009).

Hall and Harhoff (2012) argue that patent renewals come closest to estimating the true value distribution of patents. Indeed, if an assignee pays renewal fees, this means that she expects to earn at least the cost of the fee through the use of the technology in production, licensing and/or commercialization of the patent. This approach has been used by many scholars (e.g. Pakes and Schankerman 1984; Shankerman and Pakes 1986; van Pottelsberghe and van Zeebroeck 2008; Hegde and Sampat 2009). However, renewal data raises several issues. First, the information on renewal decision only becomes visible over time, a disadvantage compared to other indicators that are available more quickly, such as grant status. Second, renewal data are not readily available for all patent offices in a harmonized manner (Pakes 1986; Lanjouw et al. 1998; Bessen and Meurer 2008). Third, because renewal fees are relatively low, this approach is unable to say anything for the tail of the value distribution, where the highest-value patents lie (Hall and Harhoff 2012).

One way to look inside the tail of the distribution might be to use information on opposition (for the EPO system) and litigation (for the US), which indicate that both the applicant and the opposing party are willing to incur additional costs to safeguard their property rights (van der Drift 1989; Lanjouw and Schankerman 2001, 2004). Harhoff et al. (2003) and Harhoff and Reitzig (2004) confirm that oppositions and the value of patents are positively related and only 8% of all EPO patents—likely the highest-value ones—are opposed. Unfortunately, information on opposition and litigation is not yet consistently available for all patent offices in global databases such as PATSTAT.

The number of citations made to patents (or forward citations), initially proposed by Narin et al. (1987) and later popularized by Trajtenberg (1990), is one of the most frequent value indicators used in the literature. It is based on the fact that inventors are required to mention prior art, implying that the more a patent is cited, the more relevant it is to subsequent inventors. The number of citation-weighted patents a firm files is strongly correlated with measures of firm value based on financial market data (Harhoff et al. 1999; Lanjouw and Schankerman 2001; Hall et al. 2005; Moser et al. 2012). Interestingly, Hall et al. (2005) show that self-citations (i.e. citations made by a firm to its own patents) are actually more valued by the market than other citations. A general drawback of patent citations is that they can be used strategically by applicants (Abrams et al. 2013), introducing noise in the measure. From a practical point of view however, the wide availability of this measure in patent databases constitutes a major advantage.

Finally, patent family size, introduced by Putnam (1996), refers to the number of countries in which the applicant has sought protection for a given invention. More specifically, patent families include all the patents claiming the same priority (or set of priorities), and are usually thought of as the set of patents protecting the same invention in different countries (for a very complete review of the definition of patent families, see Martinez 2010). Putnam (1996), Harhoff et al. (2003) and van Pottelsberghe and van Zeebroeck (2008) find a positive correlation between patent value and the number of countries in which patent protection is sought for the same invention. Patent families are also at the origin of simpler indicators. Triadic families (e.g. families including patents applied for at the Japan, U.S. and European patent offices) are probably the most common one (Guellec and van Pottelsberghe de la Potterie 2004; Dernis and Kahn 2004; Aghion et al. 2016). Other indicators require patent families to include at least two triadic offices (Henderson and Cockburn 1996; Grupp 1998) or more than one patent office (Dechezleprêtre et al. 2011). Frietsch and Schmoch (2010) propose a measure called “transnational patents”. It includes all patent families with at least a PCT application (see below) or an EPO application. The rationale behind these measures is that a patent should be more valuable if the cost associated to multiple filings has been born to acquire the protection in a large number of countries.

It is not unusual that patent families include several patents *within the same patent system* (Martinez 2010). This aspect of patent families has been relatively under-explored. Harhoff (2006) establishes a correspondence between the filing route and the structure of patent families within the European Patent System. Van Zeebroeck and van Pottelsberghe (2011a) find that the size of EP patent families (as defined by the number of national validations) and the filing of divisional applications (patent applications containing matter from a previously filed application) are positively correlated with various indicators of patent value based on citations, families, renewals and oppositions. Hegde et al. (2009) study the continuation procedures within the U.S. patent system. These procedures allow applicants to base several applications on the same priority. Empirical analysis suggests that, depending on the sectors, they may be used strategically either to strengthen the protection of valuable invention, or to obtain more patents on less important innovations.

## The law and economics of patent families

Using patents as a measure of innovation activity requires some understanding of how and why they are taken out, how they are administered, how they are enforced and how all this changes over time. Before presenting data on the structure of national and international patent families, we first review the legal and economic drivers of their formation. The decision to file several patents for the same innovation depends on the procedures presiding over international patent applications and on the legal rules prevailing in each national patent system. The structure of patent families in turn depends on patenting decisions made by innovators.

Patent families generally refer to the extension of patent protection at the international level. In the next subsection, we first review the different rules and procedures available for applicants seeking international patent protection, before examining procedures in the U.S., European and Japanese patent systems that allow for multiple patent applications. Based on interviews with industry representatives and patent lawyers, we finally highlight the strategic motives of innovators to file several patent applications on the same invention.

### Circuits for international patent applications

Under the Paris Convention, an applicant in any country has one year after the first national application to file other applications abroad. One option for applicants seeking international protection is thus to file a national application at the patent office of each relevant country within a year. However, this strategy lacks flexibility.

A more flexible option consists in filing an international patent application under the Patent Cooperation Treaty (PCT).^4^ This option has become increasingly popular over the years.^5^ The PCT route does not result in an “international patent” but opens a period of 30 months from the priority application during which it is possible to file other national applications. The advantage for applicants is thus to postpone the cost of national applications, and to delay the decision of whether to file national applications and in which countries. Once filed at a Receiving Office, the international application results in a report including a written opinion on patentability issued by an International Searching Authority (ISA) within 16 months after the first (priority) filing.^6^ This report is helpful for applicants to decide whether it would be worthwhile to file national applications. It may also be used subsequently by examiners in national patent offices—and thus save search fees for the applicants.

Another option available for inventions seeking protection in European countries is to file a European patent application under the European Patent Convention^7^ (EPC). Successful applications lead to a European patent granted by the European Patent Office (EPO). The European patent merely confers applicants the right to obtain national patents in designated countries without any additional examination, provided the required fees are paid and the patent is properly translated in the national language. By contrast with PCT applications, EP applications result in European patents that are distinct from the subsequent national patents in designated countries. Although these European patents do not confer any protection *per se*, European patent applications can be priority applications, and they frequently appear in international patent families.^8^

### Second domestic filings, divisional applications and continuations in the European, U.S. and Japanese patent systems

Patent families may include several patents not only at the international level, but also within each national patent system. These multiple patents are usually second filings, including *divisional applications* or *continuing applications*, i.e. patent applications containing matter from a previously filed application, which is claimed as its *priority*.

Under the Paris Convention, an applicant who has filed a priority application has a first general possibility to file second applications for the same invention within the (1-year) priority period. Such second filings typically claim the parent’s priority, so that the applicant can have the benefit of the filing date of the original application. This is particularly important, as it defines the point in time up to when prior art can—in general lines—be considered relevant against the application. The so called “right to priority” gives applicants some margin of time in order to decide which markets are of interest to them and to further improve the industrialisation and business case before engaging in onerous international patent procedures.

As well as the usual “right to priority” in the sense of the Paris Convention, applicants may use the longer time margins (about 30–31 months) allowed by the international processing of patent applications under the Patent Cooperation Treaty (PCT) before engaging in second filings.

As well as making second filings from “priority applications”, applicants may also file divisional applications. This is guaranteed by the Paris Convention. Divisional applications are often filed when a first patent application lacks unity of invention; that is, when it describes more than one invention. Additionally, the applicant can decide to divide a first application in several divisionals as long as the parent application is pending. Divisionals thus make it possible for the applicant to seek protection for part of the subject matter disclosed but not (or no longer) claimed in the parent application. Whilst a divisional application can be filed for any pending application up to the day preceding the mention of grant of the patent, it will retain its parent’s filing date. Although these general principles hold in all patent systems, the practical conditions of their implementation may differ from one country to another, depending for instance on how unity of invention is interpreted.^9^ Divisional applications are not necessarily imposed by examiners: they may also be used deliberately by applicants.

While divisional applications are common to all patent systems, there are also specific legal provisions in the U.S. system that can induce multiple patent filings based on the same priority. The U.S. patent system offers applicants some flexibility to file *continuing applications* on purpose. Continuations make it possible to pursue additional claims to an invention disclosed in a prior application of the same applicant. They are useful when a patent examiner allowed only part of the claims in an initial application, or when an applicant identifies new ways of claiming different embodiments of the invention. Besides continuing applications, inventors can also file *continuation*-*in*-*part* applications. In contrast to simple continuations, *continuation*-*in*-*part* applications make it possible to also protect subject matter that was not disclosed in the parent application.^10^ Moreover, the protection of claims for the added subject matter do not start from the date of filing of the priority, but from their own date of filing. The USPTO introduced a new rule in 2007 restricting inventors to filing two continuation applications for each original patent application. However, this rule has been challenged in court and was eventually withdrawn in 2009.

Japanese patent law traditionally only allows patents with a narrow scope (Ordover 1991). Although the system has been evolving in the last decades^11^ (it is now closer to the European and U.S. ones), this specificity has not disappeared. Japanese patents have long been limited to a single claim—itself sharply delimited by examiners (Ordover 1991). Applicants were allowed to file several dependent claims in 1976, and in 1988 further reforms significantly extended the number of claims that could be included in a single patent (Sakakibara and Branstetter 2001). However, applicants must still pay additional fees beyond five claims, which discourage applications for broad patents. In the mid-nineties, an average Japanese patent included less than five claims, as compared with fifteen claims for an average U.S. patent (Allison and Tiller 2003). Narrower patents in turn result in more fragmented patent applications. Cohen et al. (2002) find for example that a million U.S. dollar invested in R&D generates on average 2.8 patents in Japan, as compared to 0.6 patents in the United Sates.

### Strategies for patent routes and families

Multiple patenting is thus possible under a broad set of rules in the national, regional and international patent systems. These rules are in some cases experienced as a constraint by the applicants—for instance when an examiner partly rejects an application for lack of unity of invention. However, they also offer applicants some degree of freedom to deliberately increase the number of applications based on the same priority. Our interviews with patent attorneys and IP professionals in various sectors suggest that applicants indeed find it useful to exercise this freedom, sometimes intensively.

Although practices vary greatly—reflecting the heterogeneity of technologies, legal rules, economic contexts and strategic motives—the underlying incentives are similar to all applicants. At the heart of patenting strategies lies a trade-off between the need to secure patent protection as early as possible, and the willingness to preserve the option to adjust this protection as long as possible in an evolving environment.

Inventors have strong incentives to file a priority application as soon as possible in order to protect them against imitators. Any information leakage on the invention before the priority application would be damaging in two ways. It would enable competitors to use the invention legally, and may prevent the invention from being ever patented (since through the leakage it has become prior art).^12^ Even if the secret is well kept, there is a risk under the first-to-file rule^13^ that the patent would be granted to another inventor that files an application first. Applying for a patent alleviates these risks, as it freezes relevant prior art at the date of application, and guarantees that the patent, once granted, can be opposed to any infringer.^14^

Although inventors may want to file a priority application as early as possible, they also have reasons to delay as much as possible the moment when their patent will be granted. Early applications can take place when the invention and/or its market are not yet mature, but this induces opportunity costs for the applicant. Patent applications must be disclosed after 18 months, which may provide competitors with important information on on-going R&D. If the application is filed too early, limited patent duration may also later deprive applicants of protection while the invention is still commercially exploited. Moreover, the design of a patent granted early may not perfectly match the final version of the invention, thus facilitating circumvention. To avoid such discrepancies, applicants need to delay the moment when the patent is granted with its definitive claims. Preserving some uncertainty on the design of the claims can also be a way for them to deter competitors from developing potentially infringing technology.

The rules of the patent system give applicants various means to ease the tension between early priority applications and late patent grant. At the international level, PCT applications can be seen as an option mechanism, whereby the moderate cost of filing a first application makes it possible to wait before choosing whether to extend protection internationally, and in which countries. The 30 months delay opened by the PCT route also provides substantial flexibility to fine-tune the design of the patents that will be applied for in foreign countries.^15^

At the national or regional levels, applicants can in turn use second domestic filings, including *divisional* and *continuing* applications, to delay a patent grant. By filing a *divisional* application while the parent application is still pending, applicants can obtain a second (or possibly more) divisional patent(s) granted later, and meanwhile maintain some uncertainty on the claims. In the U.S. patent system, *continuations* and *continuations*-*in*-*part* can be filed after the examination, and aim precisely at adding more claims to a patent (Hegde et al. 2009). Filing a first application with narrow claims thus makes it possible for the applicant to obtain several patents on the same invention, thereby gradually extending the overall scope of the claims and even, in the case of continuations-in-part, the duration of the patent family.^16^ It must finally be noted that filing *divisional* and *continuing* applications is costly for the applicants, who incur the related examination and representation fees. They are thus patenting strategies used selectively by applicants depending on the economic value of the invention and the potential competition.

## A look inside patents families

In this section, we take a historical look at the use of domestic (i.e. second domestic filings) and foreign extensions in the major patent offices over the past decades. The source of our data is the Worldwide Patent Statistical Database (PATSTAT, October 2016 edition), developed by the European Patent Office (EPO). PATSTAT was first published in 2006 and is updated bi-annually. The richness of the database comes from its unique geographical coverage: it gathers data from almost all of the world’s patent offices and contains over 87 million patent documents. Consequently, it is the first truly global patent database available to the research community. It should be noted that only applications that are maintained until the publication of 18 months after the priority filing date are stored. The PATSTAT database includes information on different linkages between patent applications: PCT linkages (table 201), Paris Convention priorities (table 204), domestic priorities—e.g. continuations, continuations in part, provisionals, divisionals—(table 216), and technical relations, which are established by examiners (table 205). These various linkages are used to group patent applications into patent families, available in the DOCDB patent families table.^17^ The DOCDB table includes over 87 million patent applications grouped in 63 million families^18^ and is originally intended for examiners to search for prior art and to carry out their examination of novelty. As a consequence, each DOCDB patent family includes all the documents protecting the same invention in different patent offices and allows retrieving information on the date of first filing (priority), the priority office, the date of individual filings in each office, etc.

### Domestic priorities and domestic extensions

**Fig. 1:**
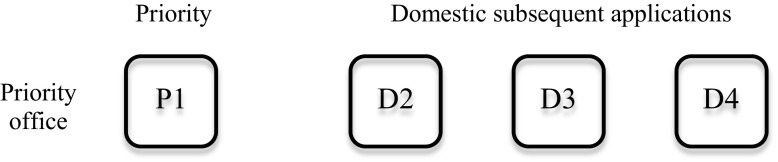
Domestic extensions

At its most basic level, a domestic patent family includes only one patent. However, it can also include one initial patent, which can be seen as the domestic priority, and a set of subsequent patent applications filed in the same office (including continuations or divisional applications), which are available in the “continuations” table of PATSTAT. An example of such a patent family is shown in Fig. 1.

Table 1 shows the number of domestic extensions per patent family in the three major patent offices for the period 1993–2010. The use of domestic extensions is most frequent at the USPTO, where the average family includes 0.24 domestic extensions, most of these being continuations. The number of continuations per parent application is highly heterogeneous. The data reveal that some patents have several hundreds of continuations (the maximum recorded in PATSTAT for a single DOCDB family being 468 continuations). Moreover, the distribution is skewed. Among patents having continuations, roughly 70% of them have only one continuation and 15% have three or more continuations. This distribution seems similar to that of the value of patents as suggested by various other value indicators (Scherer 1965; Pakes and Schankerman 1984; Pakes 1986; Griliches 1990). Alongside continuations, US patent families also include continuations-in-part and divisional applications, although used less frequently.^19^

**Table 1:** Number of domestic extensions per patent family for the main patent offices, 1993–2010

	Mean	SD	Median	Min	Max
USPTO families (*n* = 4,278,457)					
All domestic extensions	0.238	0.847	0	0	468
Continuations	0.123	0.663	0	0	468
Continuations in part	0.077	0.435	0	0	152
Divisionals	0.085	0.508	0	0	382
JPO families (*n* = 5,147,872)					
Divisionals	0.031	0.407	0	0	106
EPO families (*n* = 1,088,435)					
Divisionals	0.070	0.382	0	0	43

**Fig. 2:**
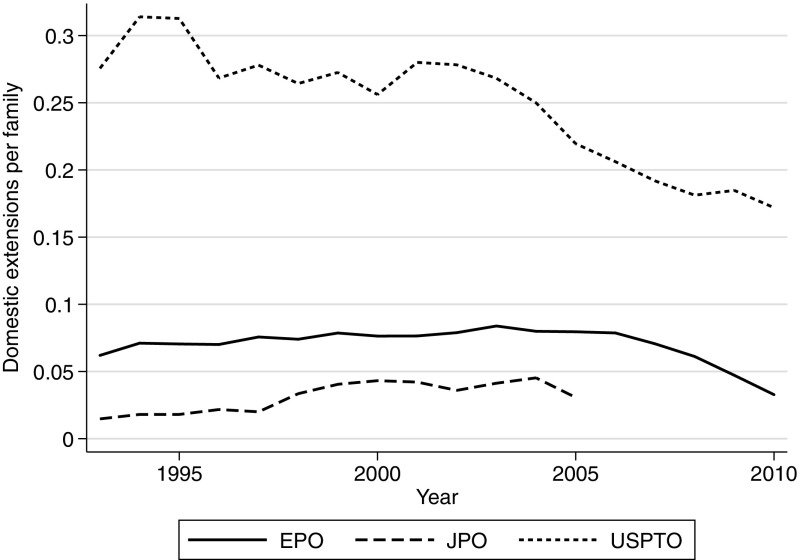
Average number of domestic extensions (divisionals and continuations) per patent family for the main patent offices (1993–2010)

Divisional applications are relatively less frequent at the EPO with a patent family having on average 0.07 divisional at the EPO and 0.03 at the JPO. Note that PATSTAT does not have a complete information on divisionals filed at the JPO (Martinez 2010), but we obtained information on JPO divisionals directly from the JPO and added those to PATSTAT’s continuation table, so that our coverage of JPO divisionals should be comprehensive, at least until 2005—for this reason, the descriptive statistics presented in Table 1 as well as in Fig. 2 are presented for the period 1993–2005 only. This result runs counter to the common belief that applicants at JPO make heavy use of divisional applications. This does not contradict the fact that JPO patent applications tend to be narrow in scope as this is not observable in domestic patent families. No formal link between patents may be reported, so that patent applications related to the same underlying invention may be represented as members of different patent families. International patent families can help us investigate this further, and we do this in the next subsection.

Figure 2 shows the evolution of the number of domestic extensions per patent family across the main offices between 1993 and 2010. The number of continuations and continuations-in-part filed at the USPTO peaked at 0.3 domestic extensions per patent family in 1994. A similar peak is observed by Hegde et al. (2009) for their analysis of continuing applications at the USPTO. We observe a decrease in the size of U.S. families after 2000. This can be related to a 1999 U.S. legislation mandating the publication of most applications 18 months after their submission. This legislation indeed suppressed the possibility for applicants to use continuations in order extend the period of secrecy prior to the issue of the patent (see Hegde et al. 2009, for a more extensive discussion of the revisions of the U.S. continuations regime). There has been an increase in the use of divisional applications at EPO and JPO in the 1990s, but the numbers have remained very low in both countries compared to the USPTO.

### International patents families

**Fig. 3:**
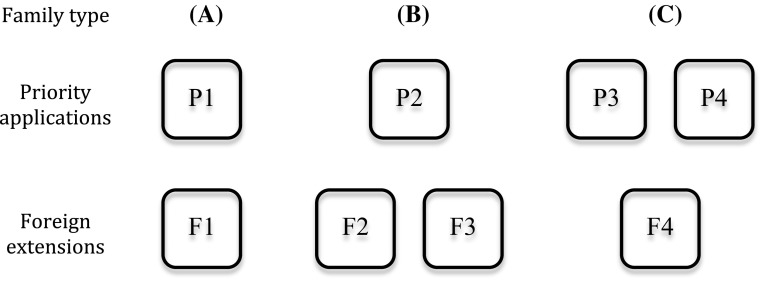
Basic two-country family structures

International patent families have numerous possible structures (see Martinez 2010, for a comprehensive overview), but a key feature is that they include patent applications filed in several patent offices. Figure 3 presents three basic linkages between priority patents filed in country X and subsequent patents filed in country Y. Case A is the simplest possible linkage: patent F1 in country Y claims priority over patent P1 in country X.^20^ P1 and F1 may have been filed through the PCT or through the Paris convention. This is the most frequent family structure. In case B, two patents (F2 and F3) in country Y claim priority over the same patent (P2) in country X. In other words, patent P2 has led to (or has been divided into) two patents F2 and F3 when transferred to country Y. F3 can also be a divisional or a continuing application from F2. Case C presents the opposite situation: patents P3 and P4 have been “merged” to form patent S4 in country Y, as S4 claims priority over both patents P3 and P4. This situation would happen for example if P4 is a continuation or a divisional patent that followed P3 but preceded F4. In that case, P3 is formally designated as the domestic priority of P4 alongside P3. In that case, the fact that F4 claims priority over both P3 and P4 means that when F4 is filed, it covers technological aspects described both in P3 and P4. P4 can also be a patent filed independently from P3—i.e. which has no formal priority link with P3.

Following the basic principle that each patent family covers a single invention, data on international patent families reveal how many priority patents cover an average invention in each country. For example, in cases A and B, the invention is covered by one priority patent, whereas in case C, it is covered by two patents. Similarly, the data also allow us to measure how many patents cover the same invention in subsequent countries. For example, in cases A and C, the invention is covered by one subsequent patent, whereas in case B, it is covered by two patents.

We now look only at patent families including applications in at least two patent offices and compare the number of patents in origin and destination countries in international families. For every DOCDB international patent family, we count the number of patent applications filed in the priority country and the number of patent applications filed in each destination country. We use information on application date, Paris convention linkages and PCT linkages to determine the priority office (in other terms, the “originating” office) of each DOCDB family.^21^ This allows us to compare the average number of patents covering a single invention in priority and subsequent offices. For example, we find that patent families originating from the US (i.e. having a USPTO priority patent) in 1993^22^ contain on average 1.66 priority patents and only 1.15 patents in each subsequent patent office. In contrast, the average family originating from Japan in that same year contains on average 1.37 priority patents, and 1.17 patents in each subsequent patent office.

Table 2 presents the average number of priority and subsequent patents per international family between 1993 and 2010 for patents *originating* from the USPTO, JPO and EPO. Three findings stand out from Table 2. First, for all three offices, the number of priority patents per patent family always turns out to be higher than the average number of subsequent patents in the destination countries. This result also applies to continuing and divisional applications, which are more frequent on average in priority offices than in subsequent offices. Second, the number of subsequent patents in the destination countries is close to one (and this has hardly changed between 1993 and 2010)., In brief, multiple applications typically take place as a first step in the priority country, while inventors usually file close to a unique foreign patent when they seek protection in other offices. Third, divisional and continuing applications are only one of the ways to cover an invention by multiple filings in the priority office. In most cases, when several patent applications are filed in the priority office, there exists no formal link between them. It is only when the patent application is filed in other offices that it becomes apparent that multiple applications in the priority office relate to the same invention. This can happen for example because the foreign patent claims priority over two initial patents as in case C of Fig. 3.

**Table 2:** Average number of patents per international family for the main patent offices, average 1993–2010

	Mean	SD	Median	Min	Max
Families originating at USPTO (*n* = 832,615)					
Applications in priority office	1.89	1.46	1	1	468
Of which divisionals	0.17	0.67	0	0	134
Of which continuations-in-part	0.14	0.64	0	0	152
Of which continuations	0.24	1.02	0	0	468
Average subsequent applications	1.11	0.32	1	1	29.3
Of which divisionals, continuations and CIP	0.02	0.12	0	0	10
Subsequent applications at EPO conditional on extending at EPO	1.12	0.51	1	1	48
Subsequent applications at JPO conditional on extending at JPO	1.18	0.72	1	1	61
Families originating at JPO (*n* = 477,747)					
Applications in priority office	1.40	1.62	1	1	319
Of which divisionals	0.14	1.06	0	0	94
Average subsequent applications	1.14	0.44	1	1	103.5
Of which divisionals, continuations and CIP	0.09	0.39	0	0	102.5
Subsequent applications at EPO conditional on extending at EPO	1.19	1.01	1	1	383
Subsequent applications at USPTO conditional on extending at USPTO	1.10	0.49	1	1	26
Families originating at EPO (*n* = 168,083)					
Applications in priority office	1.67	0.86	1	1	43
Of which divisionals	0.07	0.40	0	0	26
Average subsequent applications	1.11	0.36	1	1	37
Of which divisionals, continuations and CIP	0.04	0.22	0	0	12
Subsequent applications at USPTO conditional on extending at USPTO	1.24	0.88	1	1	90
Subsequent applications at JPO conditional on extending at JPO	1.16	1.65	1	1	147

Average subsequent applications are the average number of subsequent applications by subsequent patent office. We obtain this number by dividing the family’s total number of subsequent (foreign) applications by the number of subsequent (foreign) patent offices. Data for JPO goes until 2005 only

Table 2 also highlights differences between patent offices. We find that patent families first filed at the USPTO include 1.89 patents on average, which is higher than at the EPO (1.67) and the JPO (1.40). This reflects the specificities of national patent systems and suggests that patent families first filed in the US have a larger overall scope in their priority country than families that have a European or Japanese priority.

In Table 2, we also report the number of subsequent filings broken down by patent office of destination to explore further the heterogeneity across patent offices. Here we do not average across all patent families but instead condition on the patent family being extended in a particular office. For example, we find that patent families originating in the US and later extended at the EPO include on average 1.12 EPO patents and patent families originating in the US and later extended at the JPO include on average 1.18 JPO patents. We observe that the composition of international patent families depends on the office of priority and much less on the subsequent patent offices. For example, the average family extended at the EPO includes 1.12 EPO patents if it comes from the USPTO and 1.19 EPO patents if it comes from the JPO. Similarly, the average family extended at the JPO includes 1.18 JPO patents if it comes from the USPTO and 1.16 JPO patents if it comes from the EPO. The difference is slightly greater for USPTO patents (1.10 if coming from JPO; 1.16 if coming from EPO) although not statistically significant. In general, the number of foreign USPTO patents is greater than that of foreign EPO or JPO patents, a finding consistent with the greater use of domestic extensions at the USPTO.

**Fig. 4:**
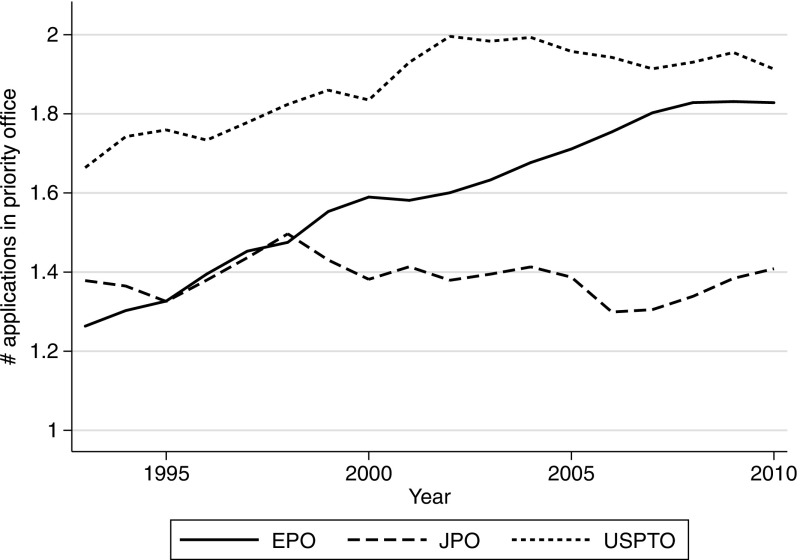
Average number of priority patents per international family for the main patent offices (1993–2010)

Figure 4 shows the number of priority patents per international family between 1993 and 2010 in the three main offices. The number of priority patents per patent family first filed at the JPO has remained stable across time. In contrast, it has been growing over time at EPO and USPTO. This suggests that the increase in the propensity to patent observed over this period (Hall 2005) in particular in the US has been at least partially driven by a growth in the size of patent families *in the priority offices*.

The finding is consistent with the applicants’ objective to obtain early protection while keeping the possibility to adjust this protection over time. As it sets the reference date for protection, the first priority is indeed filed significantly earlier (about 30 months for the PCT route) than subsequent applications in other jurisdictions. This allows applicants to adapt the scope of their patent protection to evolving technology and market condition. It is therefore not surprising that applicants make a more intensive use of available flexibilities at the priority office to file other domestic applications related to this first priority.

It is important to keep in mind that such filing strategies concern only a subset of all families, and primarily international families. We show in the next section that there are mainly used for inventions that present a strong potential, and that they can therefore be used as a statistical signal of patent value.

## Number of patent applications in the priority country and timespan of patent families as indicators of patent value

“The law and economics of patent families” and “A look inside patents families” sections show that, in the construction of patent families at the domestic and international levels, patent applications filed in the priority office and patent applications filed in subsequent (foreign) offices are qualitatively different. Patent applicants use the various rules permitted by patent systems (second domestic filings, PCT) to file multiple applications as a first step in the priority country, before filing a unique foreign patent application in other offices. These options are used to gradually extend the scope of the claims and the duration of the patent family and to fine-tune the design of the final patent that will be filed in foreign offices. However, because filing multiple patent applications is costly, we hypothesize that making use of these various options—at the domestic or international level—reflects the value of the underlying invention.

The aim of this section is to empirically validate the prediction that the use of divisional and continuing applications and more generally the number of patents filed in the priority office within a single patent family is a relevant signal of the value of priority applications. For this purpose, we empirically examine the relationship between the number of patents filed in the priority office and common measures of patents value presented in “Patents as innovation indicators” section (i.e. forward citations, triadic patents, international family size, etc.). We disaggregate patents filed in the priority office according to their type (i.e. regular patent applications, divisionals, continuations and continuations-in-part). This analysis leads us to propose two new indicators of patent value that use both the domestic and the international aspects of patent families: the total number of members in a given patent family and the timespan between the first application date and the last application date within a patent family.

### Econometric approach and descriptive statistics

In order to measure the relationship between the number of patents filed in the priority office within the patent family of priority patent *i* and various measures of patent value, we use data on all patent families originating at EPO, USPTO and JPO between 1993 and 2010.^23^ This includes both domestic-only and international patent families. Following Zeebroeck and Pottelsberghe (2011a), we estimate models of the following general form:

1 $$V_{i} = f\left( {D_{i} ,P_{i} ,X_{i} } \right)$$

where *V*_*i*_ is a measure of the value of priority patent *i*, *D*_*i*_ is the number of patents filed in the priority office belonging to the patent family of patent *i* (further disaggregated into different patent application types, see below), *P*_*i*_ is a vector of characteristics of patent *i* and *X*_*i*_ is a vector of other control variables that includes sector, applicant country and application year fixed effects.

In order to identify the priority patent *i* within each DOCDB patent family, we combine information on Paris convention linkages, PCT linkages and application date to identify the first patent filed in the priority office of each patent family. The focal patent (the unit of observation in the regressions) is the priority application *i*.

We use five different measures of patent value for the dependent variable:

- the number of forward citations received by patent *i* within 5 years from the patent publication date. We exclude from the citation count citations made by the same inventors (i.e. self-citations) and citations made by other patents from the same family (such as citations made by potential divisional and continuation applications);^24^
- the number of patent offices included in patent *i*’s DOCDB family (i.e. family size);
- whether the patent family of patent *i* includes PCT applications;
- whether patent *i* is a member of a triadic patent family (i.e. has been applied for at the EPO, JPO and USPTO);
- whether patent *i* has been granted.

The choice of these five measures of patent value is motivated by their wide use in the literature as well as by their availability across the three offices we cover.

We use different versions of *D*_*i*_, the number of patents filed in the priority office linked to priority patent *i*, depending on the patent office:

- the number of divisional applications;
- the number of continuations;
- the number of continuations-in-part;
- the total number of domestic patents, including international claimed priorities (through PCT or Paris convention), divisional applications, continuations or continuations-in-part.

We include the following characteristics of patent *i* in the vector *P*_*i*_:

- the number of inventors listed in the application;
- the number of IPC classes at 8-digit level listed on the patent;
- the number of backward citations.

Finally, the vector *X*_*i*_ includes the following control variables:

- dummy variables for each applicant country (to make things computationally feasible, we keep only the top 25 applicant countries, representing 99.7% of patent applications);
- dummy variables for 35 technological sectors as defined by Schmoch et al. (2006);
- dummy variables for the application year.

Table 3 presents descriptive statistics of the main variables of interest. The value measures have the usual skewed distributions discussed in “Patents as innovation indicators” section.

**Table 3:** Summary statistics, average 1993–2010

Variable	Mean	SD	Median	Min	Max
Dependent variables					
Forward citations	3.01	9.48	0	0	1334
Family size	1.65	2.30	1	1	66
Granted	0.39	0.49	0	0	1
Triadic	0.06	0.24	0	0	1
PCT	0.11	0.31	0	0	1
Main explanatory variables					
Number of domestic patents	1.17	0.71	1	1	468
Number of divisionals	0.04	0.36	0	0	134
Number of continuations	0.04	0.41	0	0	468
Number of continuations in part	0.03	0.28	0	0	152
Timespan	148.12	505.21	0	0	11,120
Control variables					
Number of IPC classes at 8-digit level	2.73	2.50	2	1	245
Number of inventors	2.03	1.54	1	1	76
Number of backward citations	4.58	13.14	0	0	1010

*N* = 10,336,753

We estimate Eq. (1) using maximum-likelihood estimator with a negative binomial specification when the dependent variable is a count (i.e. the number of forward citations and family size) and a probit estimator for the binary indicators (triadic, PCT and granted). We run regressions separately for the three major priority offices that we consider: EPO, JPO and USPTO.

### Number of patents in the priority country

In Table 4 we regress our five measures of patent value on the total number of domestic patents, be they international claimed priorities (through PCT or Paris convention), divisional applications, continuations or continuations-in-part. Columns (1) to (5) report results for patents originating from the EPO, columns (6) to (10) for patents originating from the USPTO and columns (11) to (15) for patents originating from the JPO. We find robust evidence that the number of patent applications filed in the priority country is positively correlated with the value of the initial priority patent, as measured by the family size, the number of citations received, triadic family, grant status and the existence of PCT members. The magnitude of the association differs across offices. For example, at the EPO, one additional patent filed in the priority office is associated with 57% greater family size, 13% more citations for the initial priority patent and increases the likelihood that the family will be triadic by 13 percentage points. The respective magnitudes are respectively 22, 13% and 2.2 percentage points for the USPTO and 22, 9% and 3.5 percentage points for the JPO.^25^

**Table 4:** Regression results, all priority patents

Originating office	(1)	(2)	(3)	(4)	(5)	(6)	(7)	(8)	(9)	(10)
Dependent variable	EPO					USPTO				
	Fam.	Cit.	Grant	Triad	PCT	Fam.	Cit.	Grant	Triad	PCT
Priority patents	0.46*** (0.00)	0.13*** (0.01)	−0.23*** (0.01)	0.54*** (0.01)	1.89*** (0.02)	0.22*** (0.00)	0.13*** (0.00)	0.02*** (0.00)	0.20*** (0.00)	0.64*** (0.00)
IPC8 classes	0.27*** (0.00)	0.27*** (0.01)	0.17*** (0.01)	0.52*** (0.01)	0.10*** (0.01)	0.43*** (0.00)	0.08*** (0.00)	0.33*** (0.00)	0.73*** (0.00)	0.22*** (0.00)
Number of inventors	0.13*** (0.01)	0.28*** (0.01)	0.12*** (0.01)	0.19*** (0.01)	0.08*** (0.01)	0.21*** (0.00)	0.57*** (0.00)	0.28*** (0.00)	0.29*** (0.00)	0.32*** (0.00)
Backward citations	−0.15*** (0.00)	0.50*** (0.01)	−0.24*** (0.00)	−0.17*** (0.00)	−0.38*** (0.01)	0.08*** (0.00)	0.66*** (0.00)	0.47*** (0.00)	0.12*** (0.00)	0.10*** (0.00)
Obs.	318,506	318,506	318,506	318,506	318,506	3,534,101	3,534,101	3,534,101	3,534,101	3,534,101
Pseudo R-square	0.128	0.100	0.082	0.195	0.607	0.105	0.122	0.335	0.277	0.353

Originating office	(11)	(12)	(13)	(14)	(15)
Dependent variable	JPO				
	Fam.	Cit.	Grant	Triad	PCT
Priority patents	0.22*** (0.00)	0.09*** (0.00)	0.03*** (0.00)	0.55*** (0.00)	0.76*** (0.01)
IPC8 classes	0.21*** (0.00)	0.56*** (0.00)	0.33*** (0.00)	0.44*** (0.00)	0.12*** (0.00)
Number of inventors	0.13*** (0.00)	0.41*** (0.00)	0.33*** (0.00)	0.32*** (0.00)	0.31*** (0.00)
Backward citations	0.18*** (0.00)	0.28*** (0.00)	0.58*** (0.00)	0.37*** (0.00)	0.55*** (0.00)
Obs.	6,484,146	6,484,146	6,484,146	6,484,146	6,484,146
Pseudo R-square	0.040	0.066	0.106	0.202	0.362

The dependent variable is the international family size in columns (1), (6) and (11); the number of forward citations received within 5 years of publication in columns (2), (7) and (12); a dummy variable equal to one if the patent was granted in columns (3), (8) and (13); a dummy variable equal to one if the patent is a member of a triadic family in columns (4), (9) and (14) and a dummy variable equal to one if the patent is a member of a PCT family in columns (5), (10) and (15). Columns (1), (2), (6), (7), (11) and (12) estimated by negative binomial maximum likelihood; columns (3)–(5), (8)–(10) and (13)–(15) estimated by probit. All columns include applicant country, sector and year fixed effects. Robust standard errors in brackets

*** Significant at 1% level; ** 5% level; * 10% level

The only remarkable exception to this pattern is that the number of patent applications filed in the priority country is negatively associated with the probability that the initial priority patent ultimately becomes granted at the EPO. This implies that, in contrast to the other offices, the average number of priority patents per patent family first filed at the EPO is significantly reduced during the examination process in this office.^26^ A possible explanation is that at the EPO divisionals are often used to save part of a pending priority application that is heading towards a refusal.

In Tables 5, 6 and 7 we disaggregate priority patents into their domestic components, i.e. divisional applications (Table 5), continuations (Table 6) and continuations-in-part (Table 7). We find consistent evidence that the number of divisional applications is positively associated with greater value of the initial priority application in all patent offices. This result confirms previous finding that parents of divisional are strongly associated with a higher patent value (van Zeebroeck and van Pottelsberghe 2011a).

**Table 5:** Regression results, divisional applications

Originating office	(1)	(2)	(3)	(4)	(5)	(6)	(7)	(8)	(9)	(10)
Dependent variable	EPO					USPTO				
	Fam.	Cit.	Grant	Triad	PCT	Fam.	Cit.	Grant	Triad	PCT
Divisionals	0.33*** (0.01)	0.10*** (0.01)	0.26*** (0.01)	0.15*** (0.01)	0.05*** (0.01)	0.07*** (0.00)	0.09*** (0.00)	0.22*** (0.00)	0.03*** (0.00)	0.01*** (0.00)
IPC8 classes	0.31*** (0.00)	0.28*** (0.01)	0.12*** (0.01)	0.57*** (0.01)	0.28*** (0.01)	0.49*** (0.00)	0.10*** (0.00)	0.32*** (0.00)	0.78*** (0.00)	0.35*** (0.00)
Number of inventors	0.16*** (0.01)	0.29*** (0.01)	0.08*** (0.01)	0.24*** (0.01)	0.23*** (0.01)	0.25*** (0.00)	0.58*** (0.00)	0.27*** (0.00)	0.32*** (0.00)	0.38*** (0.00)
Backward citations	−0.15*** (0.00)	0.50*** (0.01)	−0.22*** (0.00)	−0.20*** (0.00)	−0.40*** (0.00)	0.11*** (0.00)	0.68*** (0.00)	0.47*** (0.00)	0.15*** (0.00)	0.18*** (0.00)
Obs.	318,506	318,506	318,506	318,506	318,506	3,534,101	3,534,101	3,534,101	3,534,101	3,534,101
Pseudo R-square	0.110	0.099	0.075	0.135	0.381	0.090	0.121	0.337	0.253	0.241

Originating office	(11)	(12)	(13)	(14)	(15)
Dependent variable	JPO				
	Fam.	Cit.	Grant	Triad	PCT
Divisionals	0.07*** (0.01)	−0.12*** (0.01)	0.21*** (0.01)	0.35*** (0.01)	0.12*** (0.01)
IPC8 classes	0.22*** (0.00)	0.56*** (0.00)	0.33*** (0.00)	0.44*** (0.00)	0.15*** (0.00)
Number of inventors	0.14*** (0.00)	0.42*** (0.00)	0.33*** (0.00)	0.33*** (0.00)	0.32*** (0.00)
Backward citations	0.20*** (0.00)	0.29*** (0.00)	0.58*** (0.00)	0.40*** (0.00)	0.54*** (0.00)
Obs.	6,484,146	6,484,146	6,484,146	6,484,146	6,484,146
Pseudo R-square	0.031	0.066	0.106	0.143	0.221

The dependent variable is the international family size in columns (1), (6) and (11); the number of forward citations received within 5 years of publication in columns (2), (7) and (12); a dummy variable equal to one if the patent was granted in columns (3), (8) and (13); a dummy variable equal to one if the patent is a member of a triadic family in columns (4), (9) and (14) and a dummy variable equal to one if the patent is a member of a PCT family in columns (5), (10) and (15). Columns (1), (2), (6), (7), (11) and (12) estimated by negative binomial maximum likelihood; columns (3)–(5), (8)–(10) and (13)–(15) estimated by probit. All columns include applicant country, sector and year fixed effects. Robust standard errors in brackets

*** Significant at 1% level; ** 5% level; * 10% level

**Table 6:** Regression results, continuations applications

Originating office	(1)	(2)	(3)	(4)	(5)
Dependent variable	USPTO				
	Fam.	Cit.	Grant	Triad	PCT
Continuations	0.06*** (0.00)	0.13*** (0.00)	0.10*** (0.01)	0.02*** (0.00)	0.05*** (0.00)
IPC8 classes	0.49*** (0.00)	0.10*** (0.00)	0.32*** (0.00)	0.78*** (0.00)	0.34*** (0.00)
Number of inventors	0.25*** (0.00)	0.58*** (0.00)	0.27*** (0.00)	0.32*** (0.00)	0.38*** (0.00)
Backward citations	0.11*** (0.00)	0.67*** (0.00)	0.47*** (0.00)	0.15*** (0.00)	0.18*** (0.00)
Obs.	3,534,101	3,534,101	3,534,101	3,534,101	3,534,101
Pseudo R-square	0.091	0.122	0.335	0.253	0.241

The dependent variable is the international family size in column (1); the number of forward citations received within 5 years of publication in column (2); a dummy variable equal to one if the patent was granted in column (3); a dummy variable equal to one if the patent is a member of a triadic family in column (4) and a dummy variable equal to one if the patent is a member of a PCT family in column (5). Column (1) and (2) estimated by negative binomial maximum likelihood; columns (3)–(5) estimated by probit. All columns include applicant country, sector and year fixed effects. Robust standard errors in brackets

*** Significant at 1% level; ** 5% level; * 10% level

**Table 7:** Regression results, continuations-in-part

Originating office	(1)	(2)	(3)	(4)	(5)
Dependent variable	USPTO				
	Fam.	Cit.	Grant	Triad	PCT
Continuations-in-part	−0.05*** (0.00)	0.07*** (0.00)	0.08*** (0.00)	−0.12*** (0.01)	−0.05*** (0.00)
IPC8 classes	0.50*** (0.00)	0.11*** (0.00)	0.33*** (0.00)	0.80*** (0.00)	0.35*** (0.00)
Number of inventors	0.25*** (0.00)	0.59*** (0.00)	0.28*** (0.00)	0.33*** (0.00)	0.39*** (0.00)
Backward citations	0.12*** (0.00)	0.68*** (0.00)	0.47*** (0.00)	0.16*** (0.00)	0.19*** (0.00)
Obs.	3,534,101	3,534,101	3,534,101	3,534,101	3,534,101
Pseudo R-square	0.090	0.121	0.335	0.255	0.241

The dependent variable is the international family size in column (1); the number of forward citations received within 5 years of publication in column (2); a dummy variable equal to one if the patent was granted in column (3); a dummy variable equal to one if the patent is a member of a triadic family in column (4) and a dummy variable equal to one if the patent is a member of a PCT family in column (5). Column (1) and (2) estimated by negative binomial maximum likelihood; columns (3)–(5) estimated by probit. All columns include applicant country, sector and year fixed effects. Robust standard errors in brackets

*** Significant at 1% level; ** 5% level; * 10% level

In the US system, we find a strongly significant and positive correlation between all our measures of patent value for the priority patent and the number of continuations filed after this patent. In contrast, the number of continuations-in-part is positively associated with the number of forward citations and with grant status but is negatively associated with family size, triadic status and the presence of PCT members. These results are in line with the findings by Hegde et al. (2009) who find important differences in the relation between patent value and each of the three different types of USPTO filings considered (continuations, continuations in part and divisionals). A possible explanation may be that continuations and continuations-in-part cannot always be used as a priority for subsequent filings in foreign jurisdictions.

Our baseline results presented in Tables 4, 5, 6 and 7 account for differences in the propensity to patent not only across patent offices but also among technologies or sectors by including fixed effects for 35 technological sectors. However, the results presented above report average conditional correlations across all sectors. In order to examine the heterogeneity across sectors, we separately estimate Eq. (1) for the IT, medical and transport sectors, which cover a wide variety of patent uses. The IT sector is characterized by complex technologies with short product cycles, and by an intensive use of patents for strategic purposes (such as blocking or use in negotiations) that go beyond the mere prevention of imitation. By contrast, the medical industry is characterized by long product cycles, and uses patents mainly for protection purposes. The transportation sector is more traditional and stands in an intermediate position (Cohen et al. 2000).

Results are presented in Table 8. All columns include the previous controls (the number of IPC8 classes, the number of inventors, the number of backward citations as well as applicant country, sector and year fixed effects) but coefficients for these variables are not reported for brevity. For the same reason, we combine divisionals, continuations and continuations-in-part in a single variable (this only affects the USPTO, since the latter two types of patent applications are only available there).

**Table 8:** Regression results by sector

Originating office	(1)	(2)	(3)	(4)	(5)	(6)	(7)	(8)	(9)	(10)
Dependent variable	EPO					USPTO				
	Fam.	Cit.	Grant	Triad	PCT	Fam.	Cit.	Grant	Triad	PCT
*Sector*	IT					IT				
Priority patents	0.48*** (0.02)	0.17*** (0.02)	−0.35*** (0.04)	0.62*** (0.04)	2.03*** (0.13)	0.24*** (0.01)	0.11*** (0.01)	−0.03*** (0.01)	0.28*** (0.01)	0.77*** (0.02)
Divisionals + continuations	0.23*** (0.03)	0.10* (0.06)	0.28*** (0.05)	0.01 (0.04)	−0.01 (0.05)	0.01 (0.01)	0.12*** (0.01)	0.05*** (0.01)	−0.05*** (0.01)	−0.01 (0.01)
Obs.	10,357	10,357	10,357	10,357	10,357	48,036	48,036	48,036	48,036	48,036
*Sector*	Medical					Medical				
Priority patents	0.47*** (0.02)	0.15*** (0.02)	−0.27*** (0.04)	0.49*** (0.02)	1.87*** (0.07)	0.26*** (0.01)	0.13*** (0.01)	−0.02*** (0.00)	0.22*** (0.01)	0.82*** (0.02)
Divisionals + continuations	0.18*** (0.03)	−0.01 (0.05)	0.30*** (0.04)	−0.02 (0.04)	−0.11** (0.05)	0.05*** (0.00)	0.15*** (0.01)	0.05*** (0.01)	0.02*** (0.01)	0.07*** (0.01)
Obs.	10,812	10,812	10,812	10,812	10,812	80,131	80,131	80,131	80,131	80,131
*Sector*	Transport					Transport				
Priority patents	0.57*** (0.02)	0.11*** (0.02)	−0.16*** (0.05)	0.45*** (0.04)	2.03*** (0.09)	0.32*** (0.01)	0.09*** (0.01)	−0.04*** (0.01)	0.23*** (0.01)	0.92*** (0.02)
Divisionals + continuations	0.43*** (0.07)	0.12*** (0.02)	0.35*** (0.06)	0.25*** (0.06)	0.16*** (0.06)	0.10*** (0.01)	0.10*** (0.01)	0.06*** (0.01)	0.06*** (0.01)	0.13*** (0.01)
Obs.	9625	9625	9625	9625	9625	84,215	84,215	84,215	84,215	84,215

Originating office	(11)	(12)	(13)	(14)	(15)
Dependent variable	JPO				
	Fam.	Cit.	Grant	Triad	PCT
*Sector*	IT				
Priority patents	0.44*** (0.01)	0.15*** (0.01)	−0.05*** (0.01)	0.67*** (0.02)	0.94*** (0.03)
Divisionals + continuations	0.25*** (0.02)	−0.25*** (0.02)	0.30*** (0.02)	0.35*** (0.02)	0.14*** (0.02)
Obs.	196,863	196,863	196,863	196,863	196,863
*Sector*	Medical				
Priority patents	0.30*** (0.02)	0.07*** (0.01)	0.04*** (0.01)	0.66*** (0.03)	0.85*** (0.03)
Divisionals + continuations	0.19*** (0.03)	−0.21*** (0.04)	0.24*** (0.02)	0.44*** (0.02)	0.22*** (0.02)
Obs.	200,381	200,381	200,381	200,381	200,381
*Sector*	Transport				
Priority patents	0.07*** (0.01)	−0.03*** (0.01)	0.12*** (0.01)	0.50*** (0.02)	0.38*** (0.02)
Divisionals + continuations	0.11*** (0.01)	−0.27*** (0.03)	0.19*** (0.02)	0.55*** (0.03)	0.15*** (0.02)
Obs.	332,145	332,145	332,145	332,145	332,145

The dependent variable is the international family size in columns (1), (6) and (11); the number of forward citations received within 5 years of publication in columns (2), (7) and (12); a dummy variable equal to one if the patent was granted in columns (3), (8) and (13); a dummy variable equal to one if the patent is a member of a triadic family in columns (4), (9) and (14) and a dummy variable equal to one if the patent is a member of a PCT family in columns (5), (10) and (15). Columns (1), (2), (6), (7), (11) and (12) estimated by negative binomial maximum likelihood; columns (3)–(5), (8)–(10) and (13)–(15) estimated by probit. All columns include the number of IPC8 classes, the number of inventors, the number of backward citations as well as applicant country, sector and year fixed effects (not reported for brevity). Robust standard errors in brackets

*** Significant at 1% level; ** 5% level; * 10% level

The main takeaway messages from Table 8 are that the results presented above are robust to considering individual sectors and that there is little heterogeneity across technological fields. In all three sectors, the number of patents filed in the priority office within a patent family is positively correlated with the value of the initial priority application. The only exception is again a negative and significant correlation between the number of priority patents and the likelihood that the initial priority patent gets granted. This negative relationship stands out at the EPO, and can be observed to a lesser extent at the USPTO, for all three sectors. The magnitude of the coefficients is generally similar across sectors, except at JPO where it is smaller in the transportation sector.

Similarly, in all three sectors, the number of domestic extensions (divisionals, continuations and continuations-in-part) is positively correlated with the value of the initial priority application. This positive relationship seems particularly strong for the transportation sector at the EPO and the USPTO. By contrast, the use of divisional and continuation applications seems less strongly correlated with patent value in the IT and medical sectors at these two offices, but not at the JPO.

### Family timespan

The results from the previous section provide strong evidence that the number of patent applications filed in the priority country is a relevant signal of the value of the invention initially protected by the first priority application. Filing multiple applications allows applicants to gradually adjust the overall scope of the patent family and, with continuations-in-part, to extend their duration. At the international level, patent applicants make use of other existing options, in particular the PCT route which opens a 30-months window, to fine-tune the design of the patents that will be applied for in foreign countries.

This suggests that the timespan between the first application date and the last application date within a patent family should be strongly correlated with the value of the invention. This measure captures the use of patenting procedures by applicants to optimize the scope of patent protection over time. An advantage of this new indicator of patent value is that it incorporates both the domestic and the international aspects of patent families and can thus be calculated for both single- and multi-country patent family.

Table 9 reports the results of regressions where the five measures of patent value are regressed on the family timespan and the same set of control variables as before, including focal patent characteristics and sector, applicant country and year fixed effects. We find a remarkably consistent, strongly statistically significant, and positive correlation between the timespan of a patent family and the value of the priority patent. The result holds across all five measures of patent value (family size, citations, grant status, triadic family and PCT membership) and across the three patent offices considered. The magnitude of the coefficients is very similar across patent offices: a 10% increase in the timespan is associated with a 16–18% increase in family size and a 4–5% increase in the number of citations received.^27^ It is associated with an increased likelihood that the initial priority patent will be granted by 1–2 percentage points, an increased likelihood that the family will be triadic by 1–5 percentage points and an increased likelihood that the family will go through the PCT route by 1–3 percentage points.

**Table 9:** Regression results, patent family timespan

Originating office	(1)	(2)	(3)	(4)	(5)	(6)	(7)	(8)	(9)	(10)
Dependent variable	EPO					USPTO				
	Fam.	Cit.	Grant	Triad	PCT	Fam.	Cit.	Grant	Triad	PCT
Family timespan	0.16*** (0.00)	0.04*** (0.00)	0.06*** (0.00)	0.22*** (0.00)	0.14*** (0.00)	0.16*** (0.00)	0.05*** (0.00)	0.05*** (0.00)	0.19*** (0.00)	0.17*** (0.00)
IPC8 classes	0.19*** (0.00)	0.25*** (0.01)	0.08*** (0.01)	0.47*** (0.01)	0.17*** (0.01)	0.31*** (0.00)	0.05*** (0.00)	0.28*** (0.00)	0.66*** (0.00)	0.18*** (0.00)
Number of inventors	0.12*** (0.00)	0.28*** (0.01)	0.07*** (0.01)	0.21*** (0.01)	0.19*** (0.01)	0.14*** (0.00)	0.56*** (0.00)	0.25*** (0.00)	0.24*** (0.00)	0.30*** (0.00)
Backward citations	−0.16*** (0.00)	0.50*** (0.01)	−0.22*** (0.00)	−0.24*** (0.00)	−0.43*** (0.00)	0.01*** (0.00)	0.65*** (0.00)	0.45*** (0.00)	0.02*** (0.00)	0.07*** (0.00)
Obs.	318,506	318,506	318,506	318,506	318,506	3,534,101	3,534,101	3,534,101	3,534,101	3,534,101
Pseudo R-square	0.163	0.100	0.086	0.290	0.430	0.156	0.123	0.339	0.367	0.329

Originating office	(11)	(12)	(13)	(14)	(15)
Dependent variable	JPO				
	Fam.	Cit.	Grant	Triad	PCT
Family timespan	0.18*** (0.00)	0.04*** (0.00)	0.06*** (0.00)	0.31*** (0.00)	0.23*** (0.00)
IPC8 classes	0.06*** (0.00)	0.53*** (0.00)	0.29*** (0.00)	0.24*** (0.00)	−0.04*** (0.00)
Number of inventors	0.06*** (0.00)	0.40*** (0.00)	0.31*** (0.00)	0.28*** (0.00)	0.26*** (0.00)
Backward citations	0.10*** (0.00)	0.27*** (0.00)	0.56*** (0.00)	0.35*** (0.00)	0.53*** (0.00)
Obs.	6,484,146	6,484,146	6,484,146	6,484,146	6,484,146
Pseudo R-square	0.131	0.067	0.113	0.446	0.383

The dependent variable is the international family size in columns (1), (6) and (11); the number of forward citations received within 5 years of publication in columns (2), (7) and (12); a dummy variable equal to one if the patent was granted in columns (3), (8) and (13); a dummy variable equal to one if the patent is a member of a triadic family in columns (4), (9) and (14) and a dummy variable equal to one if the patent is a member of a PCT family in columns (5), (10) and (15). Columns (1), (2), (6), (7), (11) and (12) estimated by negative binomial maximum likelihood; columns (3)–(5), (8)–(10) and (13)–(15) estimated by probit. All columns include applicant country, sector and year fixed effects. Robust standard errors in brackets

*** Significant at 1% level; ** 5% level; * 10% level

These results suggest that the timespan of a patent family can be used, alongside other indicators, as an additional measure of patent value. The clear advantages of this measure are its availability for all types of patent families and its reliability across all major patent offices.

## Comparing innovative output based on patent counts

### The distribution of patents by type of family

The data allows us to determine the distribution of patents according to the type of family they belong to. Table 10 presents this distribution for the main patent offices. We focus on the most recent period (2000–2010). Following Martinez (2010) and others, we refer to patents filed in only one country and that are the only member of their patent family as singletons. We distinguish between four categories: (1) Domestic members of international families with a domestic priority (i.e. the priority and its second filings if any); (2) Domestic members of international families with a foreign priority (for example, a US patent extended from a Japanese priority); (3) Domestic members of domestic families (excluding singletons); (4) Singletons (i.e. patent families with only one member).

**Table 10:** Distribution of patents with respect to family type by country of priority (2000–2010)

	Domestic members of international families with domestic priority (%)	Domestic members of international families with foreign priority (%)	Domestic members of domestic (single office) families (excluding singletons) (%)	Domestic singletons (%)
Country of priority				
US	35.4	31.4	20.1	13.1
Japan	22.7	13.5	6.7	57.1
EPO	14.5	81.9	1.1	2.4
China	2.3	23.3	2.5	71.8

The proportion of patents that are taken out to foreign patent offices is strikingly small in China, compared to that of Japan or the US. The share of EPO patents for which the EPO is the priority office is relatively low. Indeed, patent applicants in European countries usually tend to first file a patent in their domestic patent office before going to the EPO, so that most patents filed at the EPO have a foreign priority. The percentage of patents with a foreign priority is significant in China, with 23%. In contrast, it is only 13% in Japan. Strictly domestic families represent 64% of patents filed at the JPO^28^ and 33% of patents filed at the USPTO. Over 70% of Chinese patents are singletons—patents that are followed neither by continuations nor by foreign applications. This suggests that the average value of patents filed at the Chinese patent office by local inventors is low. In contrast, singletons are very seldom at the EPO by the very nature of this office.

### International patent counts based on patent families with at least two patents

Patent counts are used widely to measure the innovative output of a country. How do innovative outputs based on patent counts vary according to the different types of patenting behaviour found so far?

We investigate this question in Table 11. We use various methods to calculate the world’s share of patents originating from different countries and regions and compare the numbers obtained with total R&D expenditures. We assume for simplicity that inventors always file a patent in their home patent office,^29^ so that for example a patent filed directly at JPO with no foreign priority is considered a Japanese invention.^30^ Since most applications at the EPO have foreign priorities, we restrict our analysis of Europe to Germany, as it is the largest European country in terms of patenting.

**Table 11:** Share of world’s innovation output by country of priority according to various criteria (2000–2010)

	(1)	(2)	(3)	(4)	(5)
	Share of 2000–2010 world innovation based on…				
	Number of patents	Number of families			Total R&D exp.
	All domestic patents (incl. singletons and continuations)	All domestic and intern. families	Only international families	Int. families and domestic families excl. singletons	
China	17.3	23.3	2.5	3.4	9.5
Germany	5.2	5.1	12.0	10.2	8.0
Japan	20.4	29.7	27.3	25.1	14.9
Russia	2.1	3.0	0.2	0.3	2.3
USA	21.1	13.8	26.1	32.7	35.6

*Source*: authors’ calculations from the PATSTAT database (patents and families) and OECD 2012 Factbook (R&D expenditures)

In column 1 we start by simply counting the total number of patents filed in each patent office, excluding patents previously filed in another country (i.e. claiming a foreign priority). This indicator mirrors the worldwide count of priority applications proposed by De Rassenfosse et al. (2013). With this measure China represents 17.3% of the world’s innovation output, Japan 20.4% and the USA 21.1%. These figures are significantly higher than those obtained by looking at total R&D expenditures for Japan and China but much lower for the US (see last column of Table 11).

We know however from the previous investigation that applicants at USPTO have a stronger propensity to file several patents on a single invention than applicants at JPO. Examining patent families allows us to move from a patents count to an inventions count. The advantage of counting the number of families is that this method controls for the differences in the propensity to file multiple applications for a single invention between patent offices. The result of this method can be seen in columns 2 and 3 of Table 11. In column 2, we count all patent families. As US applicants file a great number of continuations and divisionals, the share of US falls from 21% of the world’s innovation output to only 14%. The share of Chinese and Japanese innovations in the world’s innovation output increases further. The explanation for these results is that this count includes all patent families, included those filed in a single office and never extended internationally. These patents are generally of lower value. As shown in column 3, excluding purely domestic families changes the results significantly. China and Russia fall dramatically, while the performance of the US and of Germany significantly improves. Focusing only on international families, China accounts for only 2.5% of the world’s innovation. Similarly, Japan’s output decreases from 30 to 27%. The US’s share of world innovation increases from 14 to 26%.

One drawback from considering only international families is that it does not take into account inventions that have a very high value in the priority country but have simply not been transferred abroad. Golf clubs are a good example. Most patents are filed only in the US, since it is by far the largest market for golf products in the world. Being filed only in the US does not mean that the invention is of low value. In order to overcome this problem, we calculate patent counts based on the number of patent families that include at least two patents—whether these patents are filed in one or in several countries. This method includes inventions of high value protected in only one country. The results obtained with this method are shown in column 5. Interestingly, they are very close to those obtained when using R&D expenditures. This suggests that the method may provide the most accurate patent-based assessment of innovation output out of the five measures presented in Table 11. It is possible, however, that such an indicator may over-represent USPTO domestic families because continuations can only be filed in the US as well as international families made of a national EPC domestic priorities and an EPO application, because of the functioning of the EPC system. These issues should be to be taken into account in further research.

## Conclusion

This paper offers an investigation into the structure of patent families. In particular, we analyse characteristics of patent families that have been so far neglected in the literature: the number of patents protecting the invention in each patent office and the time between the first and last filing within a patent family.

We find that these characteristics of patent families reflect the maturation process of innovations. Applicants indeed face a trade-off between the pre-emption of patent protection at an early stage of the innovation development and the fine-tuning of patent protection as the innovation matures and its market potential becomes clearer. We show that national patent systems and international procedures offer various types of flexibilities that allow inventors to reconcile these objectives through sequential patent applications. Multiple applications typically take place in a first step in the country of priority, while inventors seek protection in other countries later in the maturation process by usually filing a unique patent per foreign jurisdiction.

Our empirical results also suggest that the average number of patents protecting innovations vary by priority offices and across time, reflecting the specificities of the different patent systems. In particular, we find that divisional, continuing and continuations-in-part applications are more frequently used at the USPTO and (for divisionals only) at the EPO, and that patents are less likely to be granted at the EPO in those cases.

These findings have several implications with regards to patent-based statistics. We derive two new indicators: the number of patents filed in the priority country within a family, and the time elapsed between the first filing date and the last filing date within a patent family. We show that both of them correlate with a number of widely established measures of the value of the initial priority patent, and can therefore provide robust proxies to measure this value. Compared to the size of international patent families, the advantage of these measures is that they provide a metric available for all inventions, including those that are never patented abroad.

Such metrics can also prove useful to better control for the difference in the propensity to use patents between countries. We suggest in particular that basing cross-country comparisons of innovative activity on the number of patent families that include at least two patents (whether filed in one or in several countries) might be preferable to counting only the number of international families. Indeed, this method controls for the difference in the propensity to file multiple applications for a single invention between patent offices, and excludes singletons of very low value while including high-value domestic inventions.

## Footnotes

1 See Martinez (2010) and Martinez (2011) for the different implementations of this principle.

2 A priority right is a time-limited right, triggered by the first filing of a patent application for an invention. The applicant is allowed to file subsequent applications in other countries for the same invention effective as of the date of filing the first (priority) application.

3 Reitzig (2004), Sapsalis and van Pottelsberghe de la Potterie (2007) and Greenhalgh and Rogers (2007) survey the earlier empirical literature on patent value.

4 The Patent Cooperation Treaty (PCT) is an international patent law treaty concluded in 1970. It provides a unified procedure for filing patent applications to protect inventions in each of its contracting states. Over 140 member countries are members.

5 In 2007, 50.3% of total non-resident filings were filed through the PCT procedure, compared to 25.4% in 1995 (World Patent Report 2009).

6 It is optionally followed by a preliminary examination, performed by an International Preliminary Examining Authority (IPEA).

7 The European Patent Convention (EPC) was signed in 1973. It provides a legal framework for the granting of European patents, via a single, harmonized procedure before the European Patent Office.

8 In 2012, EU member states agreed to create a European unitary patent that will guarantee supranational protection for inventions in 26 countries across Europe. The unitary patent will enter into force upon ratification of a Unified European patent court by at least 13 EU member states, including Germany, France and the United Kingdom.

9 The practice related to the filing of divisional applications at the EPO was for instance clarified in 2007: the EPO Enlarged Board of Appeal held that divisional applications containing subject-matter extending beyond that of the earlier application could be amended to remove the deficiency even though the initial application was no longer pending. In 2010, a time-limit was introduced for voluntary division of the parent application by the applicant. This rule was removed in 2014, thereby aligning the EPO with the practice of USPTO and JPO.

10 Applicants are required to disclose *continuation-in-part* applications because they change the description of the invention. *Continuing* applications are not disclosed for they are based exactly on the same invention as the priority application.

11 Partly in compliance with the TRIPS (Trade-Related Aspects of Intellectual Property Rights) agreement.

12 In some countries, a grace period may however allow for public disclosure of an invention (under certain conditions) without affecting the validity of a subsequent patent application up to a certain delay.

13 In a first-to-file system, the right to the grant of a patent for a given invention lies with the first person to file a patent application for protection of that invention, regardless of the date of actual invention. This rule is now used in almost all countries. In the United States, the first-to-invent rule prevailed until 2013, when the first to file system was adopted with the America Invents Act.

14 Note that the incentives to delay grant date probably vary across technological fields and types of applicants. They are likely to be weaker in fields characterized by quick technological upgrading such as IT and for start-up businesses, for whom possessing a granted patent could increase the likelihood of receiving external funding (see Farre-Mensa et al. 2016).

15 It is even possible to file another (post-PCT) application in the priority country.

16 For example, inventor Jerome Lemelson filed a series of continuations over thirty years to get a very broad patent on bar code readers. This patent was issued in 1984, long after bar code readers had become an integral part of the U.S. economy. Jerome Lemelson was then able to collect over a billion dollars in license fees from large companies using bar code readers. Lemelson had taken advantage of the facts (both abolished now in US patent law) that (1) a patent, once issued, lasted for 17 years from the date of issue, and (2) applications were kept secret until granted (Lemley and Moore 2004).

17 According to the PATSTAT Data Catalog, applications sharing the same DOCDB family identifier “most probably share exactly the same priorities (Paris Convention or technical relation) as in table TLS204_PRIOR_APPLN, TLS205_TECH_REL and tls216_APPLN_CONTN.” The terms “most probably” stem from the fact that patent examiners may add a patent application to a DOCDB family even if it hasn’t claimed priority over one of the family’s patents if they consider that the application does in fact belongs to the family (for example because they share the same technical content and the same applicant, or because they result from PCT applications).

18 Obviously, many families include only one patent, hence the very large total number of families.

19 Note that some domestic extensions are both recorded as continuation and as continuation-in-part, so that the sum of continuations, continuations in part and divisional applications is greater than the total number of extensions.

20 We cannot check that patents P1 and S1 have exactly identical contents. This would require the help of a patent expert and this work is clearly out of reach given the million records included in the data set. However, we can reasonably assume that the technological content of S1 and P1 is roughly similar.

21 For around 5% of patent families, it is not possible to determine with certainty which office is the priority office. We discard these families from our working dataset.

22 When a patent family includes several priority applications, we use the earliest filing year of the set of priorities as the family’s priority year.

23 For the USPTO, only granted patents are included up to 2000.

24 Some citations may obviously be missing from the PATSTAT database. We do not know of any investigation of the extent of this potential problem. Generally speaking, since most citations come from domestic patents and we focus on focal patents filed at EPO, USPTO and JPO for which the data coverage in Patstat is good, we do not worry too much about this issue. In the presence of classical measurement error, this would lead to attenuation bias, and our point estimates should be seen as a lower bound of the true estimates.

25 If we count forward citations at the level of the family instead of at the level of the priority patent, the results are robust for EPO and USPTO (coefficients are respectively 0.25*** and 0.19***) but not for JPO, for which we find a small but negative coefficient (−0.01***).

26 Note that the incentives to delay grant date probably vary across technological fields and types of applicants. They are likely to be weaker in fields characterized by quick technological upgrading such as IT and for start-up businesses, for whom possessing a granted patent could increase the likelihood of receiving external funding (see Farre-Mensa et al. 2016).

27 We use the log of timespan as the explanatory variable to mitigate the effect of outliers, so that the coefficient can be interpreted as an elasticity.

28 Recall that we cannot identify with certainty all divisional applications filed at the JPO.

29 De Rassenfosse et al (2013) show that the home office attracts the majority of priority filings. In 2000, 96.9% of US inventors first filed at the USPTO. The proportion was 98.7% for Chinese inventors, 99.3% for Japanese inventors, 91.7% for German inventors and 99.1% for Russian inventors.

30 The same methodology applies if we have information on the inventor's country of residence.

## References

[CR1] Abrams, D. S., Akcigit, U., & Popadak, J. (2013). *Patent value and citations: Creative destruction or strategic disruption?* (No. w19647). National Bureau of Economic Research Working Paper.

[CR2] Aghion P, Dechezleprêtre A, Hemous D, Martin R, Van Reenen J (2016). Carbon taxes, path dependency and directed technical change: Evidence from the auto industry. Journal of Political Economy.

[CR3] Allison JR, Tiller EH (2003). The business method patent myth. Berkeley Technology Law Journal.

[CR4] Bessen J, Meurer MJ (2008). Patent failure: How judges, bureaucrats, and lawyers put innovators at risk.

[CR5] Cohen WM, Goto A, Nagata A, Nelson RR, Walsh JP (2002). R&D spillovers, patents and the incentives to innovate in Japan and the United States. Research Policy.

[CR6] Cohen, W. M., Nelson, R. R., & Walsh, J. P. (2000). *Protecting their intellectual assets: Appropriability conditions and why US manufacturing firms patent (or not)*. (No. W7552) National Bureau of Economic Research Working Paper.

[CR7] De Rassenfosse G, Dernis H, Guellec D, Picci L, van Pottelsberghe de la Potterie B (2013). The wordwide count of priority patents: A new indicator of inventive activity. Research Policy.

[CR8] Dechezleprêtre A, Glachant M, Haščič I, Johnstone N, Ménière Y (2011). Invention and transfer of climate change mitigation technologies: A global analysis. Review of Environmental Economics and Policy.

[CR9] Dernis H, Guellec D, van Pottelsberghe B (2001). Using patent counts for cross-country comparison of technology output. STI Review (OECD).

[CR10] Dernis, H., & Khan, M. (2004). *Triadic patent families methodology*. OECD Science, Technology and Industry working paper 2004/2, Directorate for Science, Technology and Industry, OECD, Paris. www.oecd.org/sti/working-papers.

[CR11] Eaton J, Kortum S (1999). International technology diffusion: Theory and measurement. International Economic Review.

[CR12] Farre-Mensa, J., Hegde, D., & Ljungqvist, A. (2016). *The bright side of patents*. (No. w21959) National Bureau of Economic Research working paper.

[CR13] Freeman. (1982). The economics of industrial innovation, University of Illinois at Urbana-Champaign’s Academy for Entrepreneurial Leadership Historical Research Reference in Entrepreneurship.

[CR14] Frietsch, R., & Schmoch, U. (2006). Technological structures and performance as reflected by patent indicators. National System of Innovation in Comparison, pp. 89–105.

[CR15] Frietsch R, Schmoch U (2010). Transnational patents and international markets. Scientometrics.

[CR16] Gambardella A, Harhoff D, Verspagen B (2008). The Value of European Patents. European Management Review.

[CR17] Greenhalgh C, Rogers M (2007). The value of intellectual property rights to firms and society. Oxford Review of Economic Policy.

[CR18] Griliches Z (1990). Patent statistics as economic indicators: A survey. Journal of Economic Literature.

[CR19] Grupp H (1997). The links between competitiveness, firms’ innovative activities and public R&D support in Germany: An empirical analysis. Technology Analysis & Strategic Management.

[CR20] Grupp H (1998). Foundations of the economics of innovation. Theory, measurement and practice.

[CR21] Guellec D, van Pottelsberghe de la Potterie B (2000). Applications, grants and the value of patent. Economic Letters.

[CR22] Guellec, D., & van Pottelsberghe de la Potterie, B. (2004). *Measuring the globalisation of technology*. An approach based on patent data, CEB working paper 04-13.

[CR23] Hall B (2005). Exploring the patent explosion. Journal of Technology Transfer.

[CR24] Hall B, Harhoff D (2012). Recent research on the economics of patents. Annual Review of Economics.

[CR25] Hall B, Jaffe A, Trajtenberg M (2005). Market value and patent citations. RAND Journal of Economics.

[CR26] Hall B, Ziedonis R (2001). The patent paradox revisited: An empirical study of patenting in the U.S. semiconductor industry, 1979–1995. RAND Journal of Economics.

[CR27] Harhoff, D. (2006). Patent constructionism: Exploring the microstructure of patent portfolios. *Presentation prepared for the EPO/OECD conference on patent statistics for policy decision making,* Vienna, October 23–24, 2006. http://academy.epo.org/schedule/2006/ac04/_pdf/monday/Harhoff.pdf.

[CR28] Harhoff, D. (2009). *Economic cost-benefit analysis of a unified and integrated European patent litigation system*. Final report to the European Commission. http://ec.europa.eu/internal/_market/indprop/docs/patent/studies/litigation/_system/_en.pdf.

[CR29] Harhoff D, Narin F, Scherer FM, Vopel K (1999). Citation frequency and the value of patented innovation. Review of Economics and Statistics.

[CR30] Harhoff D, Reitzig M (2004). Deterinants of opposition against EPO patent grants—The case of biotechnology and pharmaceuticals. International Journal of Industrial Organization.

[CR31] Harhoff D, Scherer FM, Vopel K (2003). Citations, family size, opposition and the value of patent rights. Research Policy.

[CR32] Hegde D, Mowery DC, Graham S (2009). Pioneers, submariners, or thicket-builders: Which firms use continuations in patenting?. Management Science.

[CR33] Hegde D, Sampat B (2009). Examiner citations, applicant citations, and the private value of patents. Economics Letters.

[CR34] Henderson R, Cockburn I (1996). Scale, scope and spillovers: The determinants of research productivity in ethical drug discovery. The RAND Journal of Economics.

[CR35] Jaffe A, Lerner J (2004). Innovation and its discontents: How our broken patent system is endangering innovation and progress, and what to do about it.

[CR36] Lanjouw J, Pakes A, Putnam J (1998). How to count patents and value intellectual property: The uses of patent renewal and application data. The Journal of Industrial Economics.

[CR37] Lanjouw J, Schankerman M (2001). Characteristics of patent litigation: A window on competition. The Rand Journal of Economics.

[CR38] Lanjouw J, Schankerman M (2004). Patent quality and research productivity: Measuring innovation with multiple indicators. The Economic Journal.

[CR39] Lemley M, Moore K (2004). Ending abuse of patent continuations. Kimberly A. Moor Boston University Law Review.

[CR40] Martinez C (2010). Insight into different types of patent families.

[CR41] Martinez C (2011). Patent families: when do different definitions really matter?. Scientometrics.

[CR42] Moser, P., Ohmstedt, J., & Rhode, P. (2012). *Patent citations and the size of innovation—Evidence from hybrid corn*. Stanford University working paper.

[CR43] Narin F, Noma E, Perry R (1987). Patents as indicators of corporate technological strength. Research Policy.

[CR44] OECD (2009). Patent statistics manual.

[CR45] Ordover JA (1991). A patent system for both diffusion and exclusion. The Journal of Economic Perspectives.

[CR46] Pakes A (1986). Patents as options: Some estimates of the value of holding European patent stocks. Econometrica.

[CR47] Pakes A, Schankerman M, Griliches Z (1984). The rate of obsolescence of patents, research gestation lags, and the private rate of return to research resources. R&D, patents and productivity.

[CR48] Pavitt K (1982). R&D, patenting and innovative activities: A statistical exploration. Research Policy.

[CR49] Pavitt K (1985). Patent statistics as indicators of innovative activities: Possibilities and problems. Scientometrics.

[CR50] Putnam, J. (1996). *The value of international patent rights*. Ph.D. Thesis. Yale University Press.

[CR51] Reitzig M (2004). Improving patent valuations for management purposes—Validating new indicators by analyzing application rationales. Research Policy.

[CR52] Sakakibara M, Branstetter L (2001). Do stronger patents induce more innovation? Evidence from 1988 Japanese patent law reforms. The Rand Journal of Economics.

[CR53] Sapsalis E, van Pottelsberghe de la Potterie B (2007). The institutional sources of knowledge and the value of academic patents. Economics Innovation and New Technology.

[CR54] Schankerman M, Pakes A (1986). Estimates of the value of patent rights in European Countries during the post-1950 period. The Economic Journal.

[CR55] Scherer F (1965). Firm size, market structure, opportunity, and the output of patented innovations. The American Economic Review.

[CR56] Schmoch, U. (2004). The technological output of scientific institutions. In *Handbook of quantitative science and technology research: The use of publication and patent statistics in studies of S&T systems* (pp. 717–731). Springer, Dordrecht.

[CR57] Schmoch U, Rammer C, Legler H (2006). National systems of innovation in comparison: Structure and performance indicators for knowledge societies.

[CR58] Squicciarini, M., Dernis, H., & Criscuolo, C. (2013). *Measuring patent quality: Indicators of technological and economic value*. OECD Science, Technology and Industry working papers, 2013/03, OECD Publishing. doi:10.1787/5k4522wkw1r8-en.

[CR59] Tong X, Frame JD (1994). Measuring national technological performance with patent claims data. Research Policy.

[CR60] Trajtenberg M (1990). A penny for your quotes: Patent citations and the value of innovations. The Rand Journal of Economics.

[CR61] Van der Drift J (1989). Statistics of European patents on legal status and granting data. World Patent Information.

[CR62] Van Pottelsberghe B, van Zeebroeck N (2008). A brief history of space and time: The scope-year index as a patent value indicator based on families and renewals. Scientometrics.

[CR63] Van Zeebroeck N (2011). The puzzle of patent value indicators. Economics of Innovation and New Technology.

[CR64] Van Zeebroeck N, van Pottelsberghe B (2011). Filing strategies and patent value. Economics of Innovation and New Technology.

[CR65] Van Zeebroeck N, van Pottelsberghe B (2011). The vulnerability of patent value determinants. Economics of Innovation and New Technology.

[CR66] Van Zeebroeck N, van Pottelsberghe de la Potterie B, Guellec D (2009). Claiming more: The increased voluminosity of patent applications and its determinants. Research Policy.

